# The Pyloric Caeca Area Is a Major Site for IgM^+^ and IgT^+^ B Cell Recruitment in Response to Oral Vaccination in Rainbow Trout

**DOI:** 10.1371/journal.pone.0066118

**Published:** 2013-06-13

**Authors:** Natalia A. Ballesteros, Rosario Castro, Beatriz Abos, Sylvia S. Rodríguez Saint-Jean, Sara I. Pérez-Prieto, Carolina Tafalla

**Affiliations:** 1 Centro de Investigaciones Biológicas, Dpto. Microbiología Molecular y Biología de la Infección, Madrid, Spain; 2 Centro de Investigación en Sanidad Animal, Valdeolmos, Madrid, Spain; Universitat de Barcelona, Spain

## Abstract

Although previous studies have characterized some aspects of the immune response of the teleost gut in response to diverse pathogens or stimuli, most studies have focused on the posterior segments exclusively. However, there are still many details of how teleost intestinal immunity is regulated that remain unsolved, including the location of IgM^+^ and IgT^+^ B cells along the digestive tract and their role during the course of a local stimulus. Thus, in the current work, we have studied the B cell response in five different segments of the rainbow trout (*Oncorhynchus mykiss)* digestive tract in both naïve fish and fish orally vaccinated with an alginate-encapsulated DNA vaccine against infectious pancreatic necrosis virus (IPNV). IgM^+^ and IgT^+^ cells were identified all along the tract with the exception of the stomach in naïve fish. While IgM^+^ cells were mostly located in the lamina propria (LP), IgT^+^ cells were primarily localized as intraepithelial lymphocytes (IELs). Scattered IgM^+^ IELs were only detected in the pyloric caeca. In response to oral vaccination, the pyloric caeca region was the area of the digestive tract in which a major recruitment of B cells was demonstrated through both real time PCR and immunohistochemistry, observing a significant increase in the number of both IgM^+^ and IgT^+^ IELs. Our findings demonstrate that both IgM^+^ and IgT^+^ respond to oral stimulation and challenge the paradigm that teleost IELs are exclusively T cells. Unexpectedly, we have also detected B cells in the fat tissue associated to the digestive tract that respond to vaccination, suggesting that these cells surrounded by adipocytes also play a role in mucosal defense.

## Introduction

Mucosal immunity in fish has recently become a broadly explored field of research, mainly busted by the need for oral vaccination strategies. Despite this, there are many details of the regulatory and functional aspects of intestinal immunity which are still unknown. Moreover, as many of the features of the mucosal immune system present in mammals such as Peyer’s patches or IgA are not found in fish, very few assumptions can be established [1].

Although the structures and segments present in the digestive tract show significant differences among the diverse teleost species, a general division into three main segments has been established and was excellently reviewed by Rombout *et al*. [1]. The first segment or foregut is where the food protein uptake appears to take place, with enterocytes acting as absorptive cells. This segment includes the esophagus and a defined stomach, present in salmonids and not clearly defined in some other fish species such as cyprinids. The second segment is characterized by a strong uptake of macromolecules and enterocytes containing large supranuclear vacuoles, contains the midgut and may include a variable number of pyloric caeca (pyloric appendages) near the pylorus. Fish caeca, present in species such as salmonids, are an adaptation to increase gut surface area, contributing to a higher macromolecule uptake than that of the rest of the digestive tract. Finally, the third segment is the hindgut in which enterocytes are thought to have an osmorregulatory function, and includes an anal region that in certain species can constitute a proper rectum separated by valves.

Some previous studies have investigated diverse properties along the teleost digestive tract such as its absorption capacity, but the importance of each gut segment in terms of immunity has not been properly addressed to date [2]–[4]. Furthermore, most studies concerning the immune responses of the digestive tract, conducted upon oral or immersion stimulation, have been focused on the second gut segment, even though it has been in many occasions misnamed as hindgut when it was really referring to the second segment [1]. These posterior segments have often been used to define what we currently know concerning the presence of lymphoid populations in the digestive tract of teleost fish. Scattered lymphocytes have been observed both in the lamina propria (LP) or residing between epithelial cells. These last cells, designated as intraepithelial lymphocytes (IELs), have been observed in different species such as rainbow trout [5], carp [6] or sea bass [7]. All these studies suggested that the IELs were Ig-negative T cells. Concerning the presence of B cells in the digestive tract, strong differences are obtained among the different species. Rather than to actual differences, it has been speculated that some of these number variations are due to technical problems, including differences in antibody affinity or reactivity in the case of immunohistochemical studies or problems in the release of B cells from the connective tissue in the case of lymphocyte isolation from the gut segments. For example, abundant numbers of IgM^+^ B cells were found in the LP of carp through immunofluorescence, while isolated leukocytes from carp intestine mainly consisted in IgM^-^ cells [6]. In rainbow trout, the numbers of IgM^+^ cells reported to date in the final gut segments has always been low [8], [9]. To the light of recent discoveries, very little attention has been paid in the past years to the role of IgM^+^ cells in the teleost digestive tract. The discovery of IgT, a novel fish-specific Ig subtype [10], also named IgZ in some species [11] and the revelation that IgT^+^ cells constituted an independent B linage specialized in mucosal immunity [8], [10], may have led to the incorrect thinking that mucosal IgM specific responses do not significantly contribute to local pathogen defense. However, even if IgT plays an important role in mucosal immunity, some teleost species, such as channel catfish, do not seem to have an IgT or IgZ-like sequence but do present a specific mucosal response [12], [13]. Therefore, it seems probable that local IgM mucosal responses play an important role together with IgT responses, as in mammals both IgA and IgM-specific plasma cells have been proved to play a combined role in mucosal immunity [14], [15].

In the present study, we have investigated the distribution of B cells through the digestive tract by both real time PCR analysis of Ig mRNA levels and immunohistochemistry in physiological conditions as well as after oral immunization. For the later, we have used a previously described strategy for the oral administration of a DNA vaccine coding for the infectious pancreatic necrosis virus (IPNV) VP2 antigen after alginate encapsulation. This vaccination strategy seemed as a good model as it is capable of inducing a systemic antibody response and protection against viral challenge [16]. Our results reveal important differences in B cell presence among the different segments of the digestive tract, and surprisingly point to the pyloric caeca region as to the segment in which the recruitment of both IgM^+^ and IgT^+^ cells is most significant. Along with this difference, we have also found important variations in the transcription levels of the secreted and membrane forms of IgM, as well as the transcription factors involved in B cell maturation, Blimp1 and Pax5. Finally, we describe the presence of B cells embedded within the fat tissue associated to the digestive tract, detecting a regulation in response to local stimulation that suggests their implication in the local immune response.

## Materials and Methods

### Ethics Statement

The experiments described comply with the Guidelines of the European Union Council (86/609/EU) for the use of laboratory animals and were previously approved by the INIA Ethics committee.

### Fish

Healthy specimens of rainbow trout (Oncorhynchus mykiss) of approximately 10–20 g were obtained from Centro de Acuicultura El Molino (Madrid, Spain). Fish were maintained at the Centro de Investigaciones en Sanidad Animal (CISA-INIA) laboratory at 14°C with a re-circulating water system, 12∶12 hours L:D photoperiod and fed twice a day with a commercial diet (Skretting, Spain). Prior to any experimental procedure, fish were acclimatized to laboratory conditions for 2 weeks and during this period no clinical signs were ever observed. In addition, two pools of 5 fish were tested by standard methods to confirm the absence of any salmonid virus by isolation using BF cells [17].

### Tissue Collection in Naïve Fish

**Figure 1:**
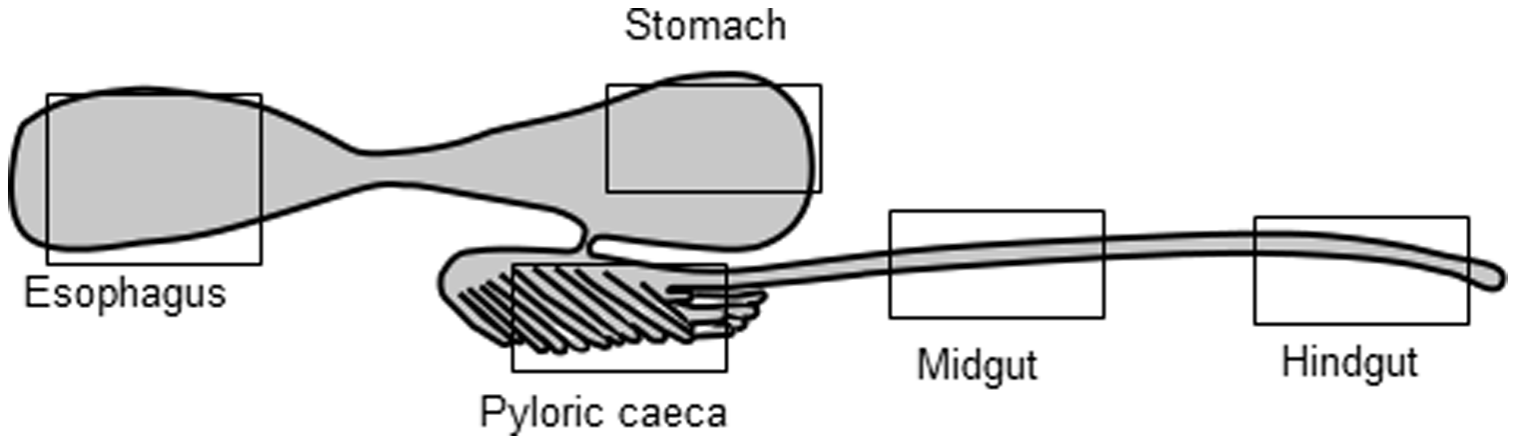
Gut segments used in this study. Schematic model illustrating the different segments in the trout digestive tract used in this study.

To characterize the B cell population along the trout digestive tract unhandled naïve trout were used. Five different segments of the digestive tract (esophagus, stomach, pyloric caeca, midgut and hindgut) were directly removed from three individual fish previously sacrificed by MS-222 overdose and were included in Bouin’s solution for further inmunohistochemical analysis. A schematic representation of the segments of the digestive tract used in this study is included in Fig. 1. To further investigate Blimp1 and Pax5 levels in the different gut segments, their levels of transcription were also analyzed in blood depleted (buffer perfused) naïve fish as well as in peripheral blood leukocytes (PBLs). For this purpose, blood was extracted from the caudal vein with a heparinized needle. Subsequently, a transcardial perfusion was conducted to remove the circulating blood from the tissues. Heart was cannulated through the ventricle into the bulbus arteriosus for perfusion with 30 ml of teleost Ringer solution pH 7.4 containing 0.1% procaine, using a peristaltic pump at a constant flow rate of ∼5 ml per min, whereas the atrium was cut to drain the blood out of the circulatory system. After perfusion, the gut segments were sampled as above (Fig. 1) and included in Trizol (Invitrogen) for further RNA extraction. Blood was diluted 10 times with of Leibovitz medium (L-15, Invitrogen) supplemented with 100 I.U./ml penicillin, 100 µg/ml streptomycin, 10 units/ml heparin and 2% fetal calf serum (FCS, Invitrogen). The resulting cell suspension was placed onto 51% Percoll density cushions which were centrifuged at 500×g for 30 min at 4°C. The interface cells were collected and washed at 500×*g* for 5 min in L-15 containing 0.1% FCS. Cells were then resuspended in Trizol for RNA extraction.

### Oral Immunization Procedure and Sampling

The pVP2 IPNV vaccine in which the IPNV VP2 gene was cloned into the pcDNA3.1/V5/His-TOPO plasmid (Invitrogen) under the control of the immediate-early CMV promoter was prepared as previously described [16], [18], [19]. The empty pcDNA3.1/V5/His-TOPO plasmid (pcDNA) was used as a control in the immunization procedures.

The procedure to encapsulate the DNA in microspheres has also been previously described [16]. Briefly, 2.5 ml of 3% (w/v) sodium alginate were mixed with 1.5 ml of pcDNA-VP2 (1 mg/ml) and the mixture stirred at 500 rpm for 10 min. This solution was then added to an Erlenmeyer flask containing 100 ml of paraffin oil and 0.5 ml Span 80, and the mixture was emulsified for 30 min at 900 rpm. Microspheres were prepared adding 2.5 ml of 0.15M CaCl_2_ drop-by-drop to the emulsion and stirring for 2 h at 900 rpm and were then collected by centrifugation at 1000×*g* for 10 min. They were washed twice with 70% ethanol, lyophilized and stored at 4°C until used.

For the immunization experiments, trout were divided into three different groups. One group was orally vaccinated with 10 µl of the vaccine microsphere suspension containing 10 µg of pVP2, while a second group received 10 µg of the pDNA empty plasmid diluted in 10 µl of a microsphere suspension. Finally, a third group received the same volume of microsphere suspension with no DNA. Vaccination was performed with an automatic pipette with a 20 µl tip which was introduced into the mouth of each trout, supporting the tip end at the entrance of the digestive tract. The water-quality parameters were maintained at optimal levels and equal in all tanks.

At day 10 post-vaccination, six fish from each group were sacrificed by MS-222 overdose and the esophagus, stomach, pyloric caeca, midgut and hindgut collected and included in Trizol for RNA extraction. This time point was chosen because previous studies had determined the highest transcription levels of the VP2 viral antigen in the midgut segment at this time (data not shown). Four additional fish in each group were sacrificed (control and vaccinated fish) and sampled for immunohistochemistry.

The levels of Ig transcription in the adipose tissue of vaccinated and mock-vaccinated fish were also studied. For this, the large accumulation of white adipose tissue located over the digestive tract was sampled in some individuals and included in Trizol for RNA extraction.

### Immunohistochemistry

Segments from the digestive tract obtained from control and orally immunized fish were fixed in Bouin’s solution for 24 h, embedded in paraffin (Paraplast Plus; Sherwood Medical) and sectioned at 5 µm. After dewaxing and rehydration, some sections were stained with hematoxylin–eosin in order to determine the levels of infiltration, apparent damages or pathological changes. A second set of sections was subjected to an indirect immunocytochemical method for detection of trout IgM and IgT using monoclonal antibodies kindly donated by Dr. Kurt Buchmann from the University of Copenhagen and Dr. Karsten Skjoedt from the University of Southern Denmark (Denmark) [20], [21]. These antibodies recognize both the membrane and the secreted forms of these Igs. Endogenous peroxidase was inhibited after rehydration by 10 min incubation in 3% H_2_O_2_ in PBS. After a heat induced epitope retrieval in Tris-EDTA buffer pH 9.0 (800 w for 5 min and 450 w for 5 min in a microwave oven) [22], the sections were pre-incubated in two different blocking solutions consisting of 2% BSA (bovine serum albumin; Sigma-Aldrich) in TBT (Tris buffer with 0.02% tween 20) at room temperature for 30 min, and 10% normal goat serum in TBT for 30 min. Then, sections were incubated with primary antibody solution overnight at 4°C. Monoclonal mouse anti-trout IgM and monoclonal mouse anti-trout IgT were used in dilutions of 1∶150 and 1∶300, respectively. Following this incubation, unbound primary antibodies were washed off using TBT. The tissue was covered with anti-mouse EnVision™ System HRP labeled secondary antibody (Dako) and left for a 30 min incubation period at room temperature. Subsequently, the tissue was washed three times with TBT and then incubated in AEC substrate [0.05M acetic acid buffer (pH 5) with 0.015% H_2_O_2_ and 0.4 g/l 3-Amino-9-ethylcarbazole (Alfa Aesar)] for 15 min and afterwards washed for 4 min in tap water. The specificity of the reactions was determined by omitting the primary antibodies. Mayer’s haematoxylin (Dako) was used as nuclear counter stain, and mounting was conducted with Aquamount (Merck). Slides were examined with an Axiolab (Zeiss) light microscope.

### cDNA Preparation

Total RNA was extracted from different gut segments or PBLs using a combination of Trizol (Invitrogen) and RNAeasy Mini kit (Quiagen). In summary, tissues were first homogenized in 1 ml of Trizol in an ice bath, two hundred µl of chloroform were then added and the suspension centrifuged at 12000×*g* for 15 min. The clear upper phase was aspirated, mixed with an equal volume of 70% ethanol in diethylpyrocarbonate (DEPC)-treated water and immediately transferred to RNAeasy Mini kit columns. The procedure was then continued following the manufacturer’s instructions, performing on-column DNase treatment. Finally, RNAs pellets were eluted from the columns in DEPC- water and stored at −80°C until used.

Four µg of RNA were used to obtain cDNA in each sample using the Bioscript reverse transcriptase (Bioline Reagents Ltd) and oligo (dT)_12–18_ (0.5 µg/ml) following the manufacturer’s instructions. The resulting cDNA was diluted and stored at −20°C.

### Evaluation of Immune Gene Expression by Real Time PCR

To evaluate the levels of transcription of different immune genes in the different segments of the digestive tract, real-time PCR was performed with a LightCycler® 480 System (Roche) using FastStart SYBR Green Master mix (Roche). Reaction mixtures containing 5 µl of 2× SYBR Green supermix, 1 µl of each primer (1 mM each) and 2 µl of cDNA template were incubated for 10 min at 95°C, followed by 40 amplification cycles (30 s at 95°C and 1 min at 60°C) and a dissociation cycle (30 s at 95°C, 1 min 60°C and 30 s at 95°C). For each mRNA, gene expression was normalized by the elongation factor 1α (EF-1α) expression in each sample and expressed as 2^−ΔCt^, where ΔCt is determined by subtracting the EF-1α Ct value from the target Ct as previously described [23]. All the primers used had already been optimized in previous studies [23]–[25]. Amplifications were performed in duplicate and negative controls with no template were always included in the reactions.

### Statistics

Prior to statistical analyses, the normal distribution of the data was checked and confirmed using the Shapiro Wilk test. To determine if there was a differential expression in the levels of gene transcription among the different groups, factorial ANOVAs were run followed by Tukey’s multiple comparison test for differences between the vaccinated group (pVP2) and the groups vaccinated with the empty plasmid (pcDNA) or mock-vaccinated. For the Pearson correlation, only the data from vaccinated fish in each segment of intestinal tract was used. The correlation was run between the levels of transcription of several immune genes and the levels of transcription of the VP2 gene. In all statistical analysis, *p* values which were less than 0.05 (*) or 0.01 (**) were considered to be significant. All statistics were run in SPSS Version 15.

## Results

### Identification of IgM^+^ and IgT^+^ Cells in the Different Gut Segments Revealed the Presence of IgM^+^ and IgT^+^ Trout IELs

**Figure 2:**
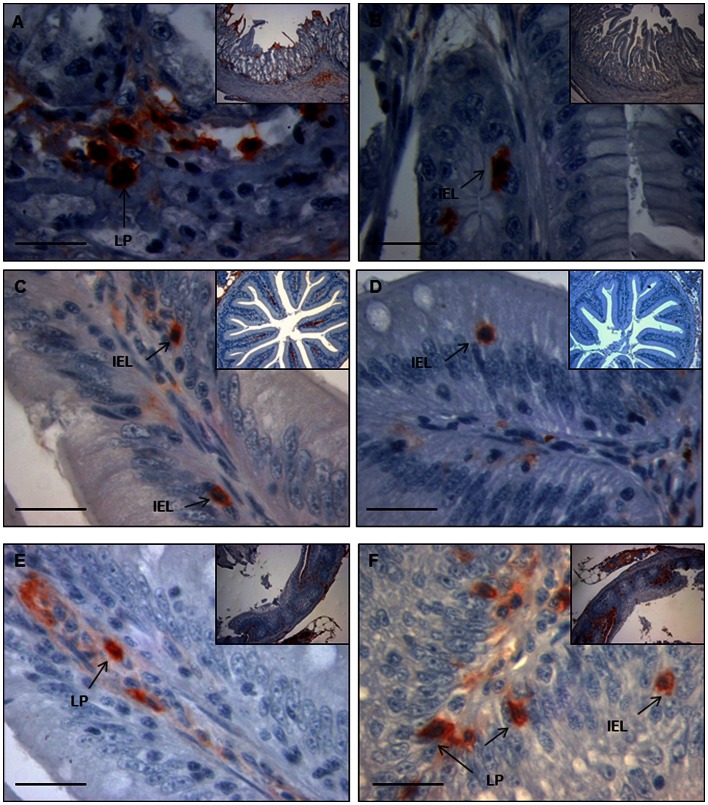
Inmunohistochemical detection of trout IgM^+^ cells and IgT^+^ cells in different segments of the digestive tract of naïve fish. Detection of IgM^+^ cells in the foregut (A), pyloric caeca (C) and midgut (E) segments revealed that IgM^+^ cells are usually located in the LP, with some IEL IgM^+^ in the pyloric caeca. Arrows show examples of LP lymphocytes (LPL) and IELs. Detection of IgT^+^ cells in the foregut (B), pyloric caeca (D) and midgut (F) segments showed that most IgT^+^ cells where located between enterocytes, as IELs. The figures show a magnification image and an insert figure of the general location in which the detail was observed. Bar: 100 µm.

Using immunohistochemistry to IgM and IgT to detect B cells in the different gut segments of naïve fish, we were able to identify numerous IgM^+^ and IgT^+^ B cells in all the segments except the stomach (data not shown). In the foregut, a strong specific reactivity to IgM was observed in the apical surface of the enterocytes, although most IgM^+^ cells were present in the loose connective tissue underlying the enterocytes, the LP (Fig. 2A). Although there was little general reactivity against IgT in the foregut, some IgT^+^ cells were clearly identified (Fig. 2B). These cells were located in the lamina epithelialis interspersed with gut epithelial cells, similarly to mammalian IELs. In the pyloric caeca, numerous IgM^+^ cells were present in the LP, although in this segment some clear IgM^+^ IELs were also identified (Fig. 2C). As observed in the foregut, IgT^+^ cells were not clearly identified in the LP of pyloric caeca, whereas most IgT^+^ cells were located between enterocytes as IELs (Fig. 2D). In the midgut region, IgM^+^ IELs were not visualized and IgM^+^ cells were located in the LP (Fig. 2E). In this segment, however, a high number of IgT^+^ cells were found both in the LP and as IELs (Fig. 2F). The IgM and IgT reactivity pattern in the hindgut was identical to that observed in the midgut (data not shown). In some cases, these IELs were small round cells similar to other IELs previously described in rainbow trout [5], whereas some other cells contained pseudopodia and a morphology that may suggest an antigen presenting role. These data confirmed that IgM^+^ and IgT^+^ cells are constitutively present in the foregut, pyloric caeca, hindgut and midgut of rainbow trout, located mainly in the LP or as IELs depending on the segment.

### VP2 is Transcribed along the Digestive Tract Upon Oral Vaccination

**Figure 3:**
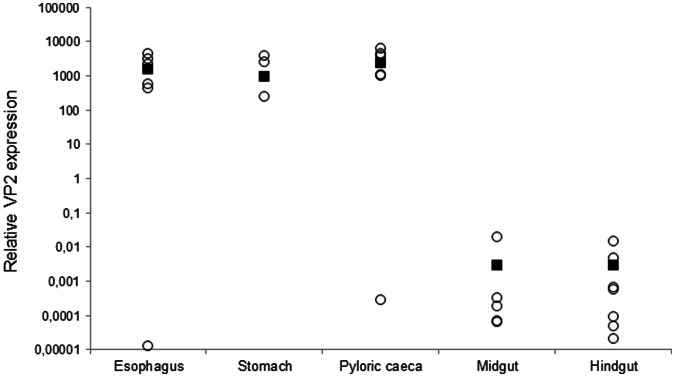
VP2 transcription in the different segments of the digestive tract of vaccinated fish. Data are shown as relative transcription levels of VP2 normalized to the transcription of the house-keeping gene EF-1α in fish orally-vaccinated with a VP2 DNA vaccine at day 10 post-vaccination. Open circles represent relative transcription levels from individual fish, whereas black squares represent mean values in each segment.

Having established the presence of tissue B cells all along the digestive tract, we proceeded to study their response to an oral vaccination protocol that had previously proved effective in controlling viral replication [16]. As a first step, we studied the levels of transcription of the IPNV VP2 gene along the different segments of the digestive tract at day 10 post-vaccination, when the VP2 levels were maximal in the pyloric caeca segment (data not shown). The highest levels of VP2 transcription were observed in the first segments, the esophagus, stomach and pyloric caeca (Fig. 3). Although still detected, the transcription of VP2 was much lower in the midgut and hindgut segments. These results reveal that the DNA vaccine is effectively taken up and transcribed by enterocytes all along the digestive tract.

### The Pyloric Caeca is the Most Responsive Segment to Oral IPNV DNA Vaccination

**Figure 4:**
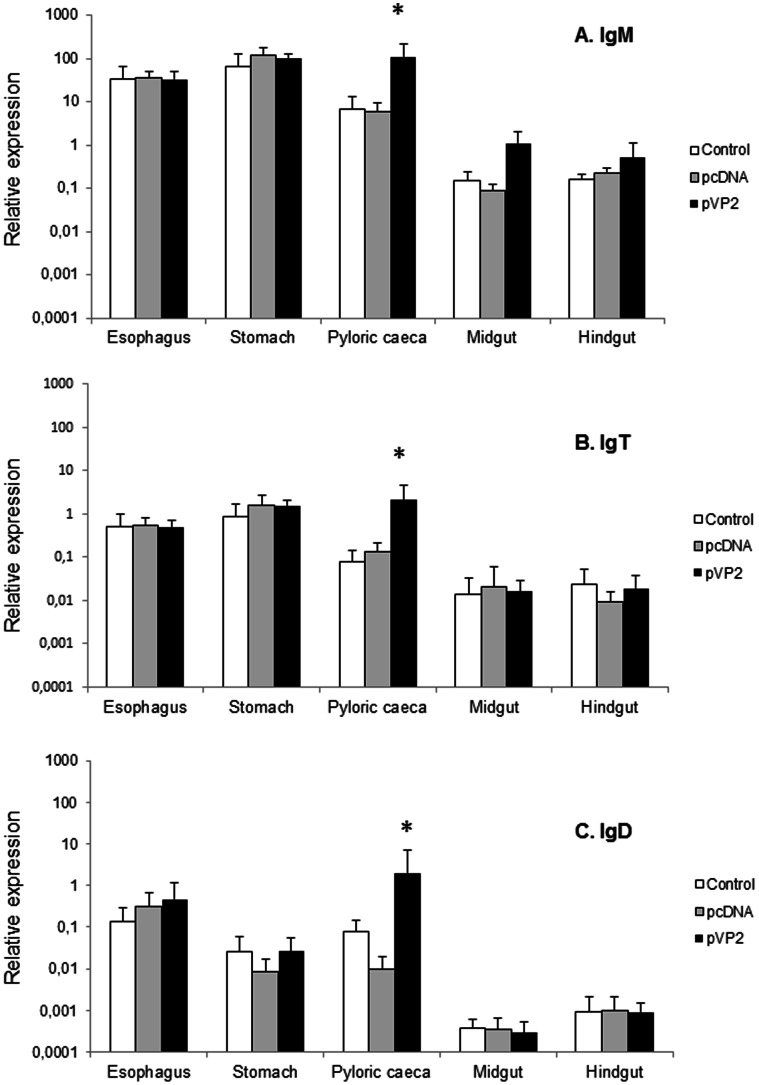
IgM, IgT and IgD modulation in response to oral DNA vaccination. Trout were orally vaccinated with 10 µl of suspension of the vaccine microspheres each containing either 10 µg of pDNA-VP2 or 10 µg of the pDNA empty plasmid diluted in 10 µl of PBS. Finally, a third group received the same volume of an empty microsphere suspension. At day 10 post-vaccination, trout were sacrificed and the different segments of the digestive tract removed for RNA extraction and analysis of immune gene transcription through real time PCR. Levels of IgM (A), IgT (B) and IgD (C) transcription in the different segments were studied through real time PCR. Data are shown as the mean relative gene expression normalized to the transcription of the house-keeping gene EF−1α ± SD (n = 6). The relative significance of differences between fish vaccinated with either the empty plasmid or empty microspheres and vaccinated fish at each segment of the digestive tract was determined through a one-way ANOVA and is shown above the bars as *.

To analyze the differences in B cell response to oral vaccination among the gut segments, we first studied the transcription levels of different Ig genes in vaccinated and control animals mock-vaccinated either with the empty plasmid or with the alginate microparticles alone. IgM, IgT and IgD transcription levels were observed all along the digestive tract in all experimental groups. However, only in the pyloric caeca region, IgM transcription was significantly higher in vaccinated fish than in control groups (either injected with the empty plasmid or with the alginate alone) (Fig. 4A). In the case of IgT and IgD transcription, again, only in the pyloric caeca region the values obtained in the vaccinated group were significantly higher than those obtained in the control groups (Fig. 4B and 4C).

**Figure 5:**
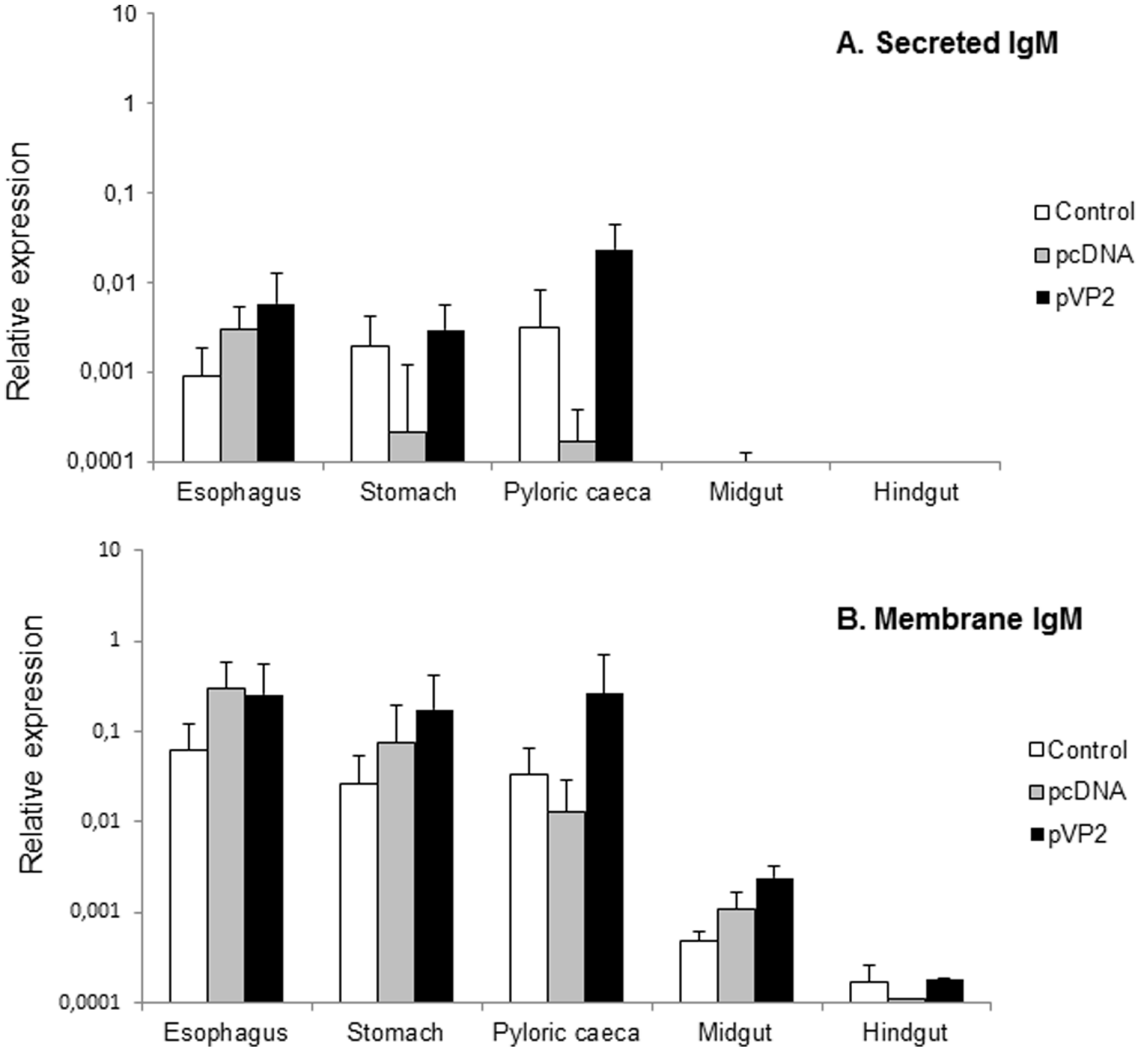
Secreted IgM and membrane IgM modulation in response to oral DNA vaccination. Trout were orally vaccinated and sampled as described in the legend of Figure 4 and levels of secreted IgM (A) and membrane IgM (B) transcription in the different segments were studied through real time PCR. Data are shown as the mean relative gene expression normalized to the transcription of the house-keeping gene EF-1α ± SD (n = 6). The relative significance of differences between fish vaccinated with either the empty plasmid or empty microspheres and vaccinated fish at each segment of the digestive tract was determined through a one-way ANOVA and is shown above the bars as *.

For IgM, specific primers have been previously designed to distinguish between the membrane form and the secreted form of IgM [25]. Using these primers, we observed that, for both the secreted and the membrane form of IgM, transcription levels were always lower in the two more distal segments, the midgut and the hindgut (Fig. 5A and 5B). In the pyloric caeca region, although we observed a higher transcription of the secreted form of IgM in vaccinated animals, the differences were not significantly different than those observed in the group mock-vaccinated with the empty alginate microspheres (Fig. 5A). Surprisingly, these values were significant in comparison to the levels detected in the group vaccinated with the empty plasmid, as there was a marked down-regulation of the transcription levels in this group. The reason for this down-regulation in the levels of transcription of secreted IgM observed in response to the empty plasmid is unknown and should be further investigated. Concerning the levels of transcription of the membrane form of IgM, we found levels of transcription significantly higher in the pyloric region of the vaccinated fish in comparison to those obtained in the control fish (Fig. 5B).

**Figure 6:**
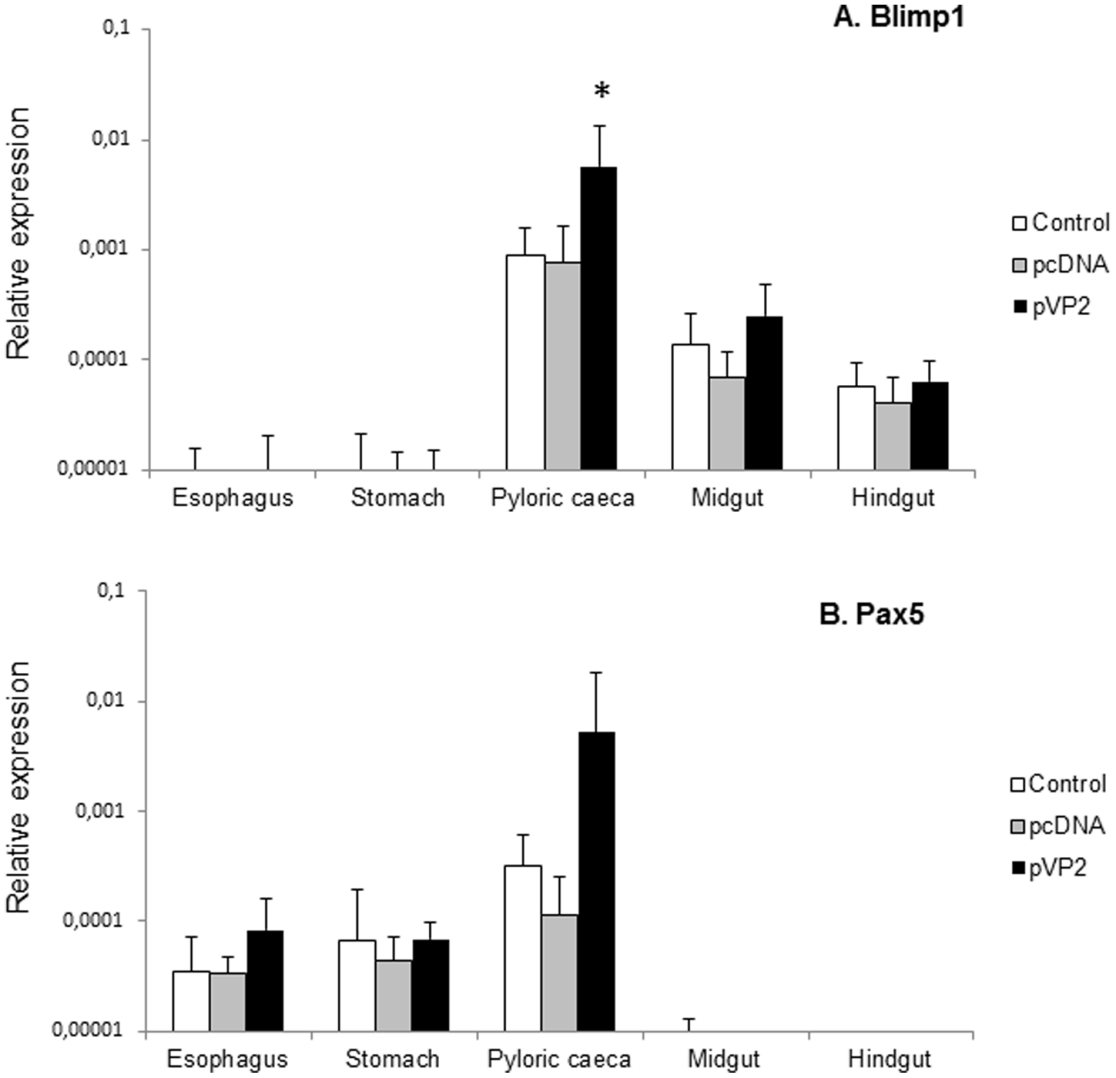
Blimp1 and Pax5 modulation in response to oral DNA vaccination. Trout were orally vaccinated and sampled as described in the legend of Figure 4 and levels of Blimp1 (A) and Pax5 (B) transcription in the different segments were studied through real time PCR. Data are shown as the mean relative gene expression normalized to the transcription of the house-keeping gene EF-1α ± SD (n = 6). The relative significance of differences between fish vaccinated with either the empty plasmid or empty microspheres and vaccinated fish at each segment of the digestive tract was determined through a one-way ANOVA and is shown above the bars as*.

To further understand a possible role of IgM^+^ B cells along the digestive tract, we also analyzed the levels of transcription of Blimp1 and Pax5. Pax5 is a B cell-specific transcription factor down-regulated through the maturation of B cells due to the induction of the transcriptional repressor Blimp1 [26]. Blimp1 transcription remained almost undetected in the esophagus and the stomach (Fig. 6A). Then the levels increased in the pyloric caeca region, to decrease again in the last two segments. In response to vaccination, it was only in the pyloric region, where differences between the vaccinated group and the control groups were significant. This same response in the pyloric caeca region was observed for Pax5 transcription (Fig. 6B); however, in this case the Pax5 transcription levels in the last two segments remain almost undetected.

### The Transcription of Genes Related to B Cell Function Correlate with Antigen Transcription

To understand whether the variations in the levels of transcription of the different immune genes analyzed were directly produced in response to vaccine transcription, we conducted a Pearson correlation analysis using the data from vaccinated animals. We found that in the pyloric caeca region there was a significant correlation between VP2 transcription and the transcription of IgM, IgT, membrane IgM and Blimp1 (Table 1). Unexpectedly, there was no correlation of VP2 transcription and IgD transcription in this segment; whereas there was a correlation between this two sets of data in the esophagus, the stomach and the hindgut segments. These data further confirm that the up-regulation in the levels of transcription of Ig variants and transcription factors observed in the pyloric caeca are directly produced by the oral vaccination procedure.

**Table 1:** Correlation of VP2 transcription with transcription of immune genes.

	Segments of the digestive tract				
Gene	Esophagus	Stomach	Pyloric caeca	Midgut	Hindgut
**IgM**	0.484	−0,413	**0.874** **	0.084	0.355
**IgT**	0.411	−0,089	**0.863** **	0.198	−0,209
**IgD**	**0.766** *	**0.984** **	0.362	−0,335	**0.742** *
**MembIgM**	0.680	−0,383	**0.799** *	−0,144	−0,228
**secIgM**	0.232	0.329	−0,002	−0,215	0.061
**Blimp1**	0.232	0.132	**0.756** *	−0,219	0.447
**Pax5**	0.060	0.278	0.374	−0,485	0.266

Pearson correlation between the levels of transcription of the VP2 gene and the transcription levels of the different immune genes studied in the different segments of the intestinal tract. N = 6.

* p<0.05;

** p<0.01.

### B Cells are Mobilized to the Pyloric Caeca Region in Response to Oral Vaccination

To confirm the results obtained through real time PCR that showed that IgM and IgT expression was up-regulated in the pyloric region in response to oral vaccination, and to establish whether this up-regulation was due to a mobilization of cells into this segment or on the other hand to an increased transcription of Ig in individual cells, we analyzed the presence of IgM^+^ and IgT^+^ cells in both vaccinated and mock-vaccinated animals by immunohistochemistry.

**Figure 7:**
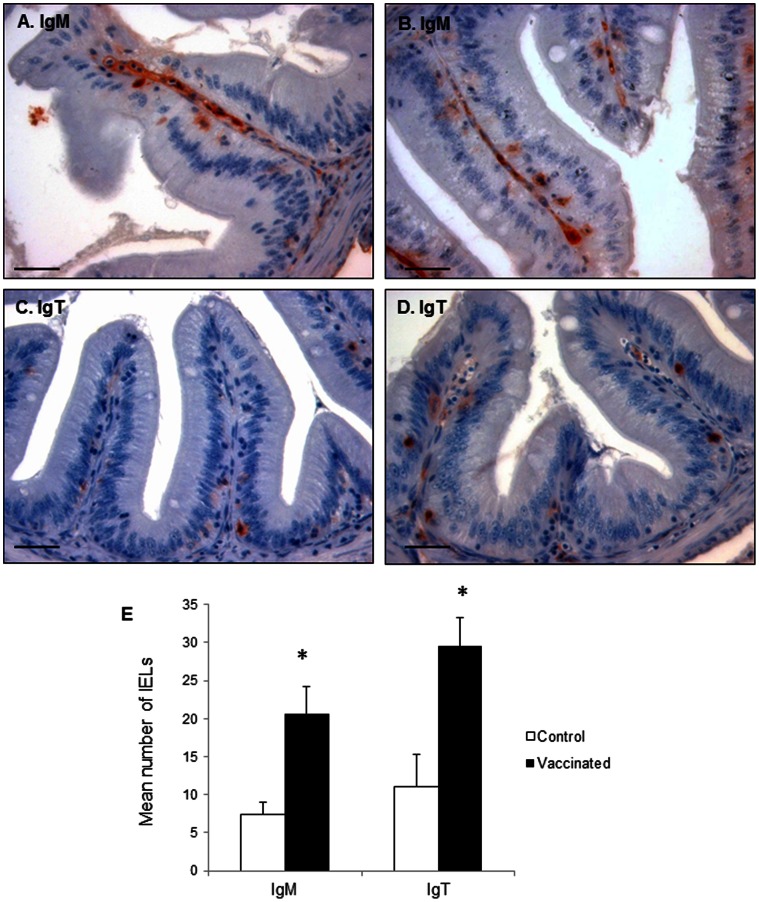
B cell detection in the pyloric caeca after oral DNA vaccination. Immunohistochemical detection of trout IgM^+^ and IgT^+^ cells in pyloric caeca of mock-vaccinated (A, C) and vaccinated (B, D) fish. Trout were orally vaccinated as described in the legend of Figure 4 and sampled at day 10 post-vaccination. Bar: 200 µm. E. Mean number of IgM^+^ and IgT^+^ IELs counted in 35 fields of control and vaccinated fish (400× magnification). The quantification was performed in triplicate in 3 individual fish per group. *Number of cells in vaccinated fish significantly higher than the number of cells in the corresponding controls.

As was described above, the pyloric caeca of naive animals contains IgM^+^ cells mainly in the LP, with some cells located between enterocytes as IELs. After vaccination, we observed that the number of IgM^+^ IELs significantly increased in this region (Fig. 7A, 7B and 7E). Although even in basal conditions most IgT^+^ cells in the pyloric caeca were found as IELs, upon vaccination, we also observed a significant increase in the number of IgT^+^ IELs, while still only some IgT^+^ cells were present in the LP (Fig. 7C, 7D and 7E).

### Blimp1 Levels and Pax5 Levels in Perfused Naïve Fish

**Figure 8:**
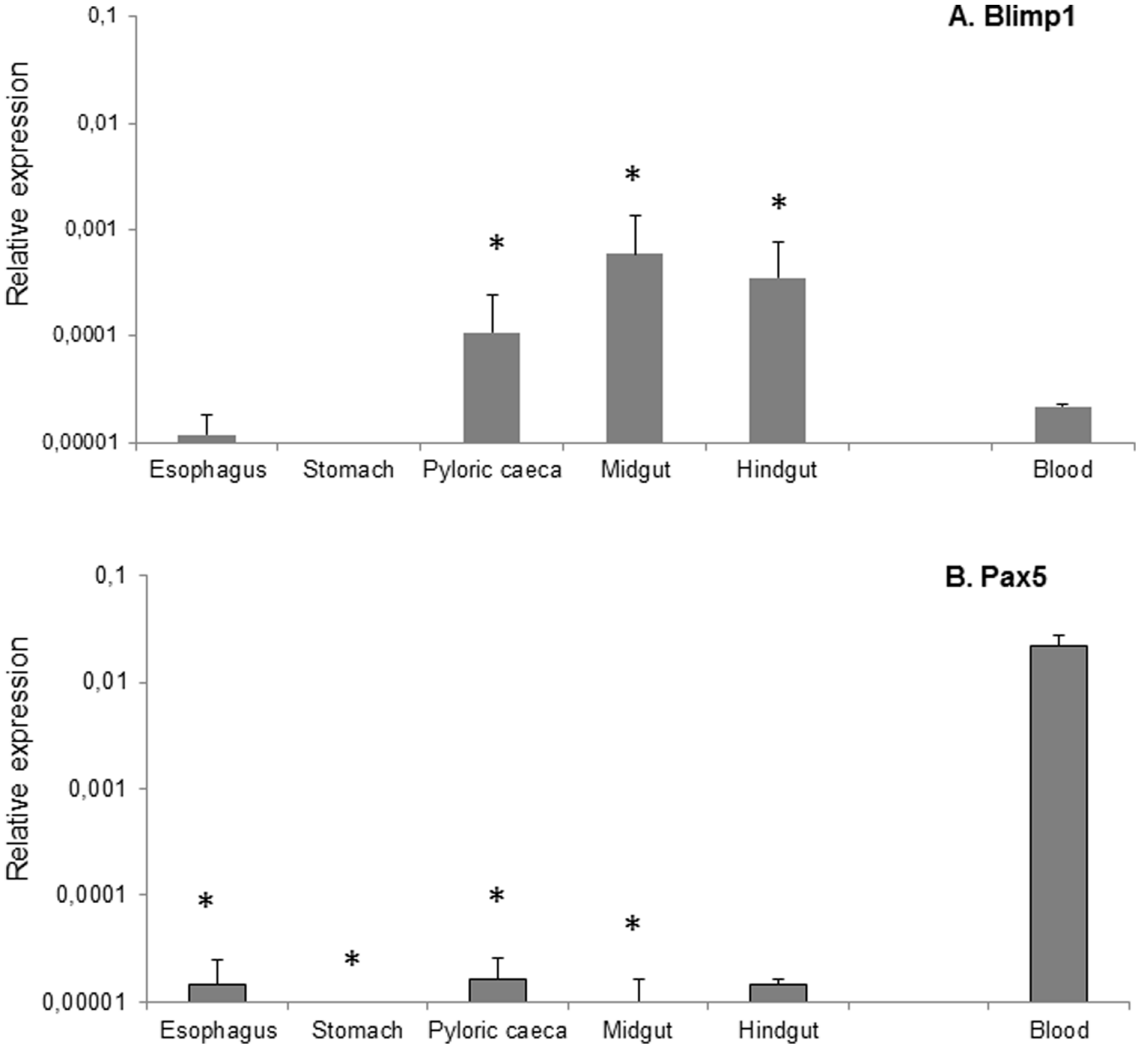
Blimp1 and Pax5 transcription in the gut of perfused fish. Constitutive levels of Blimp1 (A) and Pax5 (B) transcription in different segments of the trout digestive tract after perfusion were compared to transcription levels observed in PBLs. Data are shown as the mean gene expression relative ± SD calculated by the 2^−ΔCt^, according the formula ΔCt =  Ct gene -Ct EF-1α. * Levels of transcription in the different gut segments significantly different than transcription levels in the blood.

Given that both Blimp1 and Pax5 transcription levels were up-regulated in response to oral vaccination in the pyloric caeca, despite being *a priori* negatively correlated, we decided to further investigate the transcription of these factors. For this, the levels of transcription of Blimp1 and Pax5 in the different gut segments were studied in perfused blood-depleted naïve fish, comparing them to the levels observed in PBLs. As already observed in the vaccination trial, Blimp1 was not significantly transcribed in the foregut and stomach; however, mRNA levels were detected in the pyloric caeca, midgut and hindgut segments, at levels significantly higher than those observed in PBLs (Fig. 8A). On the other hand, Pax5 was only faintly detected in the esophagus, pyloric caeca and hindgut, whereas the levels of Pax5 transcription in PBLs were 1000 times higher (Fig. 8B). These results, together with the fact that only Blimp1 and not Pax5 levels were correlated to VP2 transcription in the pyloric caeca, suggest that B cells that respond to vaccination in the pyloric caeca have a Blimp1^high^ Pax5^low^ phenotype.

### B Cells Located in the Adipose Tissue Associated to the Digestive Tract Respond to Local Stimulation

**Figure 9:**
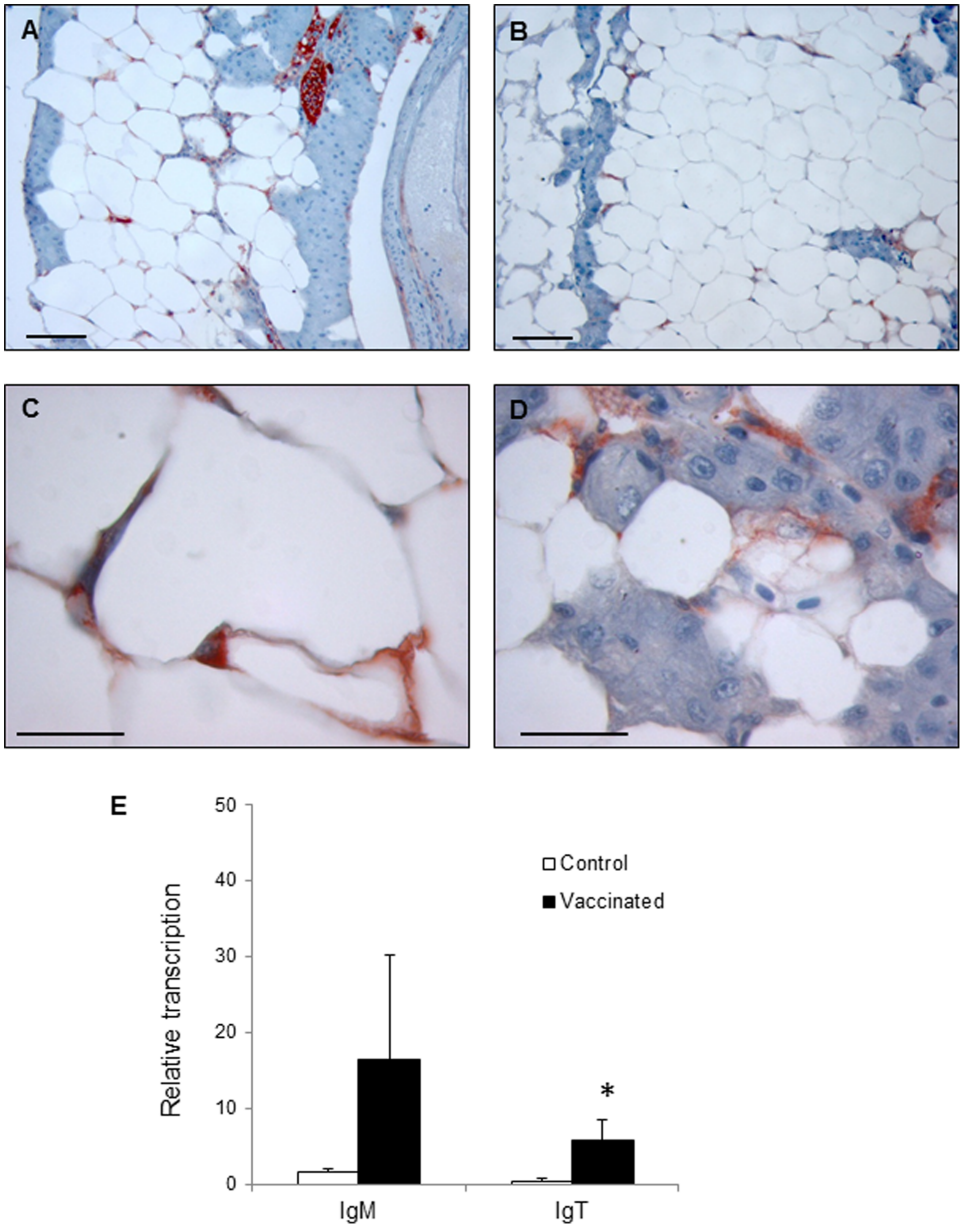
B cells in the adipose tissue surrounding the pyloric caeca. Inmunohistochemical detection of trout IgM^+^ (A, C) and IgT^+^ (B, D) cells in the adipose tissue of naïve fish. Bar: 100 µm. E. Levels of transcription of IgM and IgT estimated through real time PCR in control (mock-vaccinated with empty alginate microspheres) and vaccinated fish. Trout were orally vaccinated and sampled as described in the legend of Figure 4 and data are shown as the mean relative gene expression normalized to the transcription of the house-keeping gene EF-1α ± SD. The relative significance of differences between fish vaccinated with the empty plasmid and vaccinated fish at each segment of the digestive tract was determined through a one-way ANOVA and is shown above the bars as*.

When examining immunohistological sections of the pyloric caeca region, we observed that the fat tissue surrounding the digestive tract also contained some IgM^+^ and IgT^+^ cells (Fig. 9A–D). These IgM^+^ and IgT^+^ cells were found in the interstitial space between adipocytes. An important vascularization and endothelial cells were also observed in these adipose structures. To verify whether these immune cells embedded in the adipose tissue also responded to stimulation, we analyzed the levels of transcription of IgM and IgT in this accumulation of adipose tissue associated to the digestive tract obtained from either vaccinated or control fish. Surprisingly, the levels of transcription of both IgM and IgT increased in response to vaccination (Fig. 9E), although in the case of IgM a high variability was observed among the different fish sampled. Therefore, B cells present in the fat tissue surrounding the digestive tract were also responding to oral vaccination.

## Discussion

Although different absorption capacities had been reported along different segments of the digestive tract in different teleost species [2]–[4], their immunological properties had not been properly addressed [1]. In the current work, we have undertaken this task in relation to B cell responses, focusing on B cell mobilization and Ig transcription, and we have demonstrated that concerning B cell responses, the pyloric caeca is the most immunologically active segment of the rainbow trout digestive tract. Its high absorption capacity [1], together with the presence of IgM^+^ and IgT^+^ IELs seem as factors that could account for this higher capacity to respond to immune stimuli. Therefore, future studies dealing with immunization in salmonids should take into consideration the immune response elicited in this gut segment.

In naïve fish, both IgM^+^ and IgT^+^ B cells were detected in all the segments of the digestive tract, with the exception of the stomach. IgM^+^ cells were always found in the LP, however, in the case of the pyloric caeca, IgM^+^ IELs were also identified. On the other hand, IgT^+^ cells were mostly IELs in all segments and only sometimes they were localized in the LP in the midgut and hindgut regions. In mammals, IELs are primarily T cells with potent cytolytic and immunoregulatory capacities that because of their properties have been cataloged as a mid-way between adaptive and innate immune responses [27]. Accordingly, previous studies in rainbow trout in which IELs had been isolated from the gut, pointed out that those IELs were T cells [5], however, in that study, the pyloric caeca segment was not included nor was the search for IgT transcripts. In the current work, we demonstrate for the first time in fish that IELs include IgM^+^ and specially IgT^+^ cells. Similarly, B cells can be found as IELs in other mucosal tissues in mammals and for example IELs of human adenoids and tonsils are enriched in B cells [28]. Taking into account the levels of transcription of the transcription factors Blimp1 and Pax5 in the naïve perfused fish, we can also conclude that the B cells in the pyloric caeca, the midgut and the hindgut have a Blimp1^high^ Pax5^low^ phenotype, in comparison to what we observe in PBLs; therefore, even in basal conditions, B cells in these three segments seem to be more activated than B cells found in the blood. During this activation, Pax5 levels are reduced in part due to the induction of Blimp1 that shifts the Ig expression from membrane to the secreted form [26]. Despite this, in these posterior segments, the transcription of secreted IgM remained almost undetectable. On the other hand, an important level of transcription of secreted IgM was detected in parallel to a Blimp1^-^ phenotype in the foregut and stomach. The later could suggest the presence of B1 cells in these segments which are known to produce high levels of secreted Ig with low levels of both Blimp1 and Pax5 [29]. Furthermore, the role of Blimp1 and Pax5 in IgT^+^ B cells is also an unknown issue that should be addressed in the future, giving light to some of our present results. Future work in our group will include a further characterization of the phenotypes of these distinct B cell populations isolated from the different segments.

When fish were orally vaccinated, out of the different segments, the pyloric caeca was the only one which significantly responded to the stimulation through an increased IgM, IgT, IgD, membrane IgM, Pax5 and Blimp1 transcription. Moreover, the transcription levels of IgM, IgT, membrane IgM and Blimp1 in the vaccinated individuals strongly correlated with the transcription of the VP2 viral gene indicating that the up-regulations observed for these genes were a direct consequence of vaccine transcription. Given the fact that Pax5 and Blimp1 have been shown to be negatively correlated and that Pax5 was not correlated to VP2 transcription, we investigated Blimp1 and Pax5 transcription in perfused fish to analyze the response of tissue B cells exclusively, without any input from circulating blood. While Blimp1 levels in the pyloric caeca, midgut and hindgut segments were higher than levels detected in PBLs, the levels of expression of Pax5 in PBLs were 1000 times higher than those obtained in any gut segment. Thus, it seems probable that the up-regulation of Pax5 observed in the pyloric caeca in response to vaccination is a consequence of a higher income of blood into the tissue as a response to vaccination, whereas the up-regulation of Blimp-1 could indicate the maturation of plasma cells locally in response to vaccination.

Our results also reveal the importance of IgM in mucosal responses, challenging the actual line of thought in which IgT seemed to be the Ig subtype mainly responsible for mucosal responses [8]. As more data become available, it seems probable that IgT also plays a role in non-mucosal defense as demonstrated by the fact that IgT expansion in the spleen of virus-infected animals has been observed, revealing that these spleen IgT^+^ cells contribute to increase IgT plasma levels [30]. Our results suggest that both IgM and IgT together respond at an early stage upon mucosal immunization. It may be possible that different responses are observed as a consequence of different types of immunization, while on the other hand, it may be possible that IgT specific plasma cells take over IgM^+^ cells only at late times post-immunization, since the previous studies in which IgT^+^ cells accounted for almost all B cells in the midgut segment were performed in response to a parasite three months after the infection [8].

Finally, our studies have led us to the identification of lymphocyte cells in association with the adipose tissue that surrounds the pyloric caeca. Although the presence of immune cells in diverse adipose structures has been reported in humans and other mammalian models [31], the immune reactions and how their regulation occur in the various environments within the body have been only marginally appreciated in the past, despite the fact that there is a correlation between the presence of fat-associated lymphoid cells and inflammation in obesity [31]. Adipose tissue in mammals is generally separated into visceral and subcutaneous adipose tissue, being the visceral adipose tissue the one which is metabolically and immunologically more active [32]. In mammals, this visceral fat tissue refers to adipose within the peritoneal cavity, including depots such as the gonadal fat pad, the omentum, and the intestinal mesentery [33]. Fatty structures such as the omentum are enriched in macrophages and B cells [34], but also possess dendritic cells [35] and NKT cells [36]. In our studies, we have identified both IgM^+^ and IgT^+^ B cells in the interstitial space between adipocytes and we have demonstrated that the levels of transcription of both Ig can be regulated in response to vaccination in this area. In mammals, although B1 B cells predominate in the leukocyte clusters designated as milky spots [37], B2 conventional responses can be found in the omentum as well. For example, peritoneal immunization with bacteria provoked a strong increase in the number and size of the milky spots [34]. These milky spots and a correlated IgG production are also produced in mice lacking spleen, lymph nodes, and Peyer’s patches [38]. A more recent paper revealed that, after a gammaherpesvirus infection, immune aggregates within the omentum not only expand in size and number but also contain virus-infected cells [39]. Furthermore, in this same study, a germinal-center B cell population appeared in the omentum of infected animals with earlier kinetics and greater magnitude than that observed in the spleen. In all these experiments, the immunization was performed through intraperitoneal injection; however, not many studies have demonstrated a response of fat-associated immune cells upon oral immunization [38]. All these results point to an important role of fat-associated lymphoid structures in both innate and adaptive immune responses. Having identified these fat-associated immune cells in teleost fish, future studies should be done to determine their precise role in immunity.

In summary, in this study we describe the presence of IgM^+^ and IgT^+^B cells in the foregut, pyloric caeca, midgut and hindgut of trout, identifying IgM^+^ and IgT^+^ IELs for the first time in fish. Upon oral immunization, we have demonstrated that the number of IELs increases in the pyloric caeca region along with an up-regulation of IgM, IgT, membrane IgM and Blimp1 transcription that strongly correlates with the transcription of the vaccine antigen. Furthermore, we describe the presence of B cells in the adipose tissue associated to the pyloric caeca and their capacity to respond to an oral stimulation. All together, these results provide further insight in the characterization of mucosal B responses in teleost fish.
